# Declines in the Lethality of Suicide Attempts Explain the Decline in Suicide Deaths in Australia

**DOI:** 10.1371/journal.pone.0044565

**Published:** 2012-09-05

**Authors:** Matthew J. Spittal, Jane Pirkis, Matthew Miller, David M. Studdert

**Affiliations:** 1 Melbourne School of Population Health, The University of Melbourne, Melbourne, Australia; 2 Department of Health Policy and Management, Harvard School of Public Health, Boston, Massachusetts, United States of America; 3 Melbourne Law School, The University of Melbourne, Melbourne, Australia; University of Pennsylvania, United States of America

## Abstract

**Background:**

To investigate the epidemiology of a steep decrease in the incidence of suicide deaths in Australia.

**Methods:**

National data on suicide deaths and deliberate self-harm for the period 1994–2007 were obtained from the Australian Institute of Health and Welfare. We calculated attempt and death rates for five major methods and the lethality of these methods. Negative binomial regression was used to estimate the size and significance of method-specific time-trends in attempts and lethality.

**Results:**

Hanging, motor vehicle exhaust and firearms were the most lethal methods, and together accounted for 72% of all deaths. The lethality of motor vehicle exhaust attempts decreased sharply (RR = 0.94 per year, 95% CI 0.93–0.95) while the motor vehicle exhaust attempt rate changed little; this combination of motor vehicle exhaust trends explained nearly half of the overall decline in suicide deaths. Hanging lethality also decreased sharply (RR = 0.96 per year, 95% CI 0.956–0.965) but large increases in hanging attempts negated the effect on death rates. Firearm lethality changed little while attempts decreased.

**Conclusion:**

Declines in the lethality of suicide attempts–especially attempts by motor vehicle exhaust and hanging–explain the remarkable decline in deaths by suicide in Australia since 1997.

## Introduction

**Figure 1:**
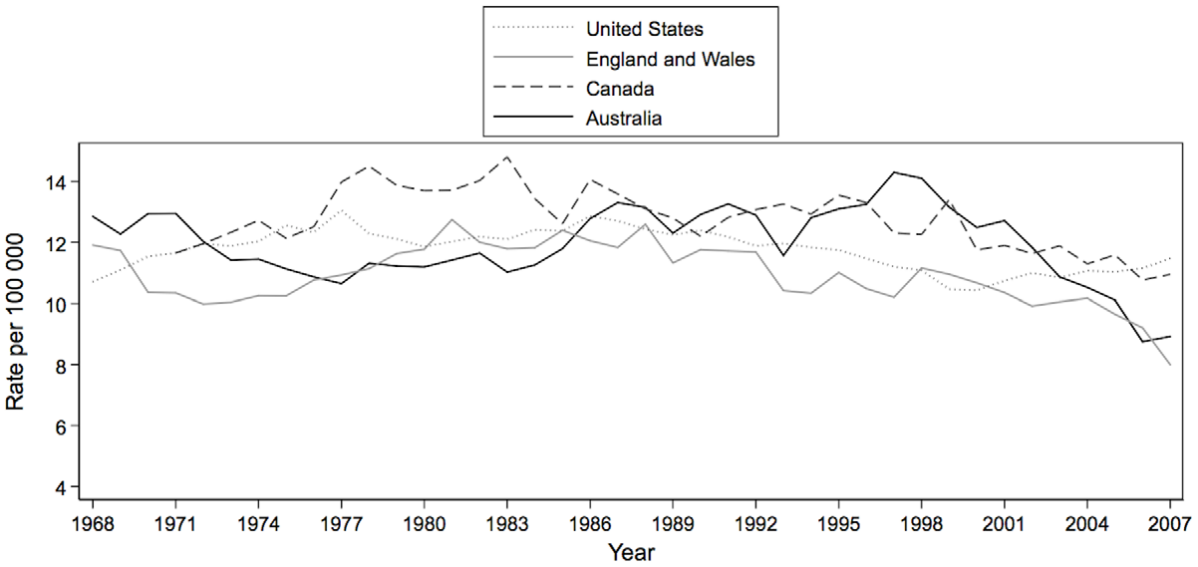
Trends in suicide deaths in United States, England and Wales, Canada, Australia, and, 1968–2007. Sources: US Centers for Disease Control, Office for National Statistics, Statistics Canada, Australian Institute of Health and Welfare.

Suicide is a major public health problem.[1], [2] The World Health Organization estimates that it accounts for nearly one million deaths per year globally and 1.9% of life years lost.[3], [4] Suicide trends differ substantially across countries, even among countries with similar socioeconomic and demographic profiles. In the United States, for example, suicide rates have declined little since the 1970s (**Figure 1**) whereas in Canada and England and Wales, rates have fallen gradually since the mid-1980s. In Australia, suicide rates were flat or modestly rising until the late 1990s, when the national suicide rate began a remarkable decline. The epidemiology of this important trend is poorly understood.

Mathematically, the number of deaths by suicide is the product of the number of all suicide attempts (both fatal and non-fatal) and the probability those attempts will result in death. Hence, any fall in suicides must be due to: (1) a decrease in the number of attempts; (2) a decrease in lethality of attempts; or (3) some combination of (1) and (2).

We analysed national data on fatal and non-fatal suicide attempts between 1994 and 2007, the latest year for which official suicide statistics are currently available. We probed the relationship between changes in the number of suicide deaths and underlying changes in method-specific attempt rates and lethality. Our main goal was to improve understanding of the decline in suicide deaths in Australia.

## Methods

### Data

We obtained annual statistics on suicide deaths and attempts from the Australian Institute of Health and Welfare (AIHW), an official custodian of vital statistics in Australia.

AIHW maintains the General Record of the Incidence of Mortality (GRIM) books.[5] The GRIM books record counts of all registered deaths in Australia by year and classify them according the World Health Organization's International Statistical Classification of Diseases and Related Health Problems (ICD). In the 9^th^ revision of the ICD, which AIHW used between 1979 and 1996, codes E950–E959 indicate suicide; in the 10^th^ revision, which AIHW used from 1997, codes X60–X84 indicate suicide. The comparability of self-harm codes across the two revisions is excellent.[5], [6] We obtained counts of all suicide deaths between 1994 and 2007 by jurisdiction (Australia is divided into 6 states and 2 territories), calendar year, and method category. At the time of our study, the latest year for which national suicide data was available was 2007; cause-of-death data for later years was either not released or was interim data only and in the process of revision by the Australian Bureau of Statistics.[7] There were five categories of methods: poisoning (E950, X60–X66, X68, X69), motor vehicle exhaust (E951, E952, X67), hanging (E953, X70), firearms (E955, X72–X75) and all other methods (E954, E956–E959, X71, X76–X84).

AIHW also compiles the National Hospital Morbidity Database, a comprehensive collection of data on hospital admissions gathered by state and territory health departments. These admissions are also classified using the ICD coding system. The 9^th^ revision was used prior to 2001, and the 10^th^ revision thereafter. We obtained counts of admissions associated with suicide attempts by state and territory, financial year, and by the same method categories as were used for deaths. Records for emergency department presentations are not included in these counts, although records for patients who are subsequently admitted to hospital are included.

Our reliance on the ICD coding of hospital admissions creates a familiar issue for population-level suicide research: reference to these acts of deliberate self-harm as suicide “attempts”, which is the terminology used throughout this report, may be somewhat over-inclusive because the intent behind these acts is not directly measured. [8] In some cases, the intent will not have been to die. However, most acts of deliberate self-harm that result in hospitalization, particularly those by methods other than poisoning and cutting, are likely to have involved some degree of intent to end life.[9], [10]

### Study Dataset

We combined the data on deaths and attempts and added mid-year population estimates for each jurisdiction[11] to produce a study dataset at the method-year level.

Several adjustments were necessary to prepare the data for analysis. Because deaths were grouped by calendar year and attempts by financial year (1 July to 31 June), we shifted the death counts forward in time; so, for example, deaths for financial year 1994/1995 were aligned with attempts in calendar year 1995 and tagged as 1995 data.

To comply with AIHW's confidentiality rules, the three jurisdictions with the smallest populations (Tasmania, the Australian Capital Territory, and the Northern Territory) had to be combined in all presentations of method-specific data. In addition, AIHW suppressed counts in 12 of the 840 cells in the study dataset (based on 6 jurisdictions × 14 years × 5 methods, for both attempt and death counts). For 6 of the suppressed cells, we used a mean of values from surrounding cells. However, the 6 other suppressed cells fell at the end of the time series and could not be reliably extrapolated (attempts by motor vehicle exhaust in South Australia and Western Australia in 2006 and 2007); hence, these cells are excluded from count data, and national rates and proportions for these two methods in 2006 and 2007 are based on data from the four remaining jurisdictions.

### Statistical Analysis

We calculated annual rates (per 100 000 persons) for suicide attempts and deaths by each method. We also calculated the lethality by method and year. Lethality, sometimes referred to as the “case fatality ratio”, was derived by dividing total number of deaths by the total number deaths and attempts, and expressed as a percentage. For one method that exhibited a large change in lethality over time, we asked a counter-factual question: how many suicide deaths would have occurred had lethality remained unchanged at its baseline level? We answered this question with a series of simple calculations: in each year, we multiplied the observed number of attempts (fatal and non-fatal) by the method's lethality in 1994.

To determine the size and significance of key trends in attempts and lethality we ran negative binomial regression analyses on the study dataset. For attempts, the outcome variable was a count of all suicide attempts in the relevant year and the predictor was a variable interacting time (in years) with the method of interest; annual population sizes were included as an offset term. For lethality, the outcome variable was a count of suicide deaths in the relevant year and the predictor was the same interaction between time and method used in the attempt analysis, with the sum of all attempts (fatal and non-fatal) in the relevant year included as an offset term. Finally, the measure used to quantify time trends was derived algebraically from the coefficients on the interaction variables in these models[12] and expressed as a rate ratio (RR) indicating the average method-specific change per year over the study period.

All analyses were undertaken in Stata 12.1.[13]

## Results

### Sample characteristics

The study sample consisted of 31 941 suicide deaths (mean of 2 282 deaths per year) and 378 977 suicide attempts (mean of 27 070 attempts per year). **Table 1** shows the distribution of these events by year, jurisdiction and method. Hanging accounted for 42% of deaths in the study period, motor vehicle exhaust (MVE) for 19%, poisoning for 13% and firearms for 11%.

**Table 1:** Counts of deaths and attempts for year, jurisdiction and method categories and lethality for the same categories.*

	Deaths	Attempts	Lethality#
Characteristic			
Year			
1994	2,287	16,797	12.0
1995	2,366	19,564	10.8
1996	2,426	21,637	10.1
1997	2,647	22,347	10.6
1998	2,639	25,853	9.3
1999	2,490	26,239	8.7
2000	2,392	27,253	8.1
2001	2,468	29,847	7.6
2002	2,326	30,492	7.1
2003	2,162	30,502	6.6
2004	2,118	31,299	6.3
2005	2,062	32,536	6.0
2006	1,771	32,307	5.2
2007	1,787	32,304	5.2
Jurisdiction			
New South Wales	10,047	124,633	7.5
Victoria	7,363	86,246	7.9
Queensland	6,640	73,769	8.3
Western Australia	3,251	44,461	6.8
South Australia	2,659	31,307	7.8
ACT, Northern Territory, Tasmania	1,981	18,561	9.6
Method †			
Hanging	13,493	9,322	59.1
Motor vehicle exhaust	6,120	7,500	44.9
Poisoning	4,216	288,160	1.4
Firearms	3,490	1,215	74.2
All other methods	4,622	72,780	6.0

* Due to AIHW suppression rules in the data provided for this study, the figures presented omit attempt and death counts for motor vehicle exhaust and firearms in Western Australia and South Australia in 2006 and 2007.

# Lethality  =  deaths/(deaths + attempts) * 100

† Categories based on coding of deaths and hospital separations according to the International Statistical Classification of Diseases and Related Health Problems (versions 9 and 10): poisoning (E950, X60–X66, X68, X69); motor vehicle exhaust (E951, E952, X67); hanging (E953, X70); firearms (E955, X72–X75); all other methods (E954, E956–E959, X71, X76–X84)

**Table 1** also shows the suicide lethality by year, jurisdiction and method. Overall, lethality declined over the study period, from 12% of all attempts resulting in death in 1994 to 5% in 2007 – a decline of 57%. Firearms were the most lethal methods, causing death in 74% of attempts; hanging (59%) and MVE (45%) were also highly lethal. By contrast, only 1.4% of the attempts by poisoning and 6.0% of the attempts by all other methods were fatal.

### Time trends by method

**Figure 2:**
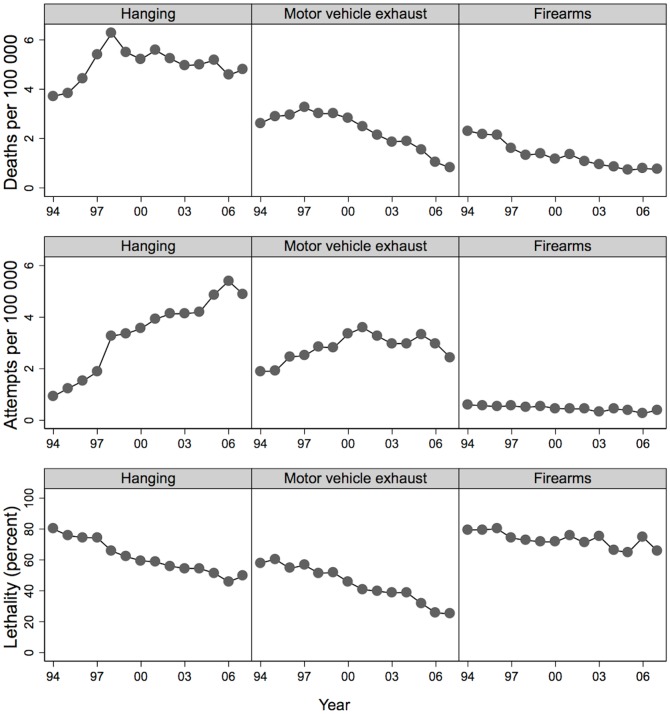
Trends in the death rate, attempt rate and lethality of hanging, motor vehicle exhaust and firearms, 1994–2007.

**Figure 2** shows trend data for hanging (left column of plots), MVE (middle column) and firearms (right column) –three methods with the highest lethality and which together accounted for 72% of all the suicide deaths. Specifically, the trends shown for each of these methods are rates of suicide deaths (top row of graphs), rates of suicide attempts (middle row) and lethality (bottom row). A decline in the number of deaths beginning in the late 1990s was apparent for all three methods, but there are major differences in how this occurred.

Deaths due to hanging increased between 1994 and 1998 and then remained flat thereafter. Behind this trend were large increases in hanging attempts (from 0.9 per 100,000 in 1994 to 4.9 per 100,000 in 2007; RR = 1.13 per year, 95% CI 1.10–1.15) and large declines in the lethality of this method (RR = 0.96 per year, 95% CI 0.956–0.965). Thus, the incidence of suicide by hanging declined in most years after 1998 because a spike in hanging attempts more than counteracted reductions in the proportion of those attempts that proved fatal.

The trends in suicides by MVE were different to hanging. MVE deaths decreased steeply after 1997. MVE attempts followed an inverted U-shaped trend–increasing, flattening, and then decreasing. This attempt trend does not explain the monotonic decline in MVE deaths. Rather, that decline was due largely to steady year-on-year decreases in the lethality of MVE attempts (RR = 0.94 per year, 95% CI 0.93–0.95).

The trends for firearms were different again. Firearm deaths decreased over the study period. This was chiefly due to decreases in attempts (from 0.6 per 100 000 in 1994 to 0.4 per 100 000 in 2007; RR = 0.96 per year, 95% CI 0.93–0.98); there was relatively little change in the lethality of those attempts (RR = 0.99 per year, 95% CI 0.98–1.00), which remained high throughout the study period.

### Impact of declines in lethality

**Figure 3:**
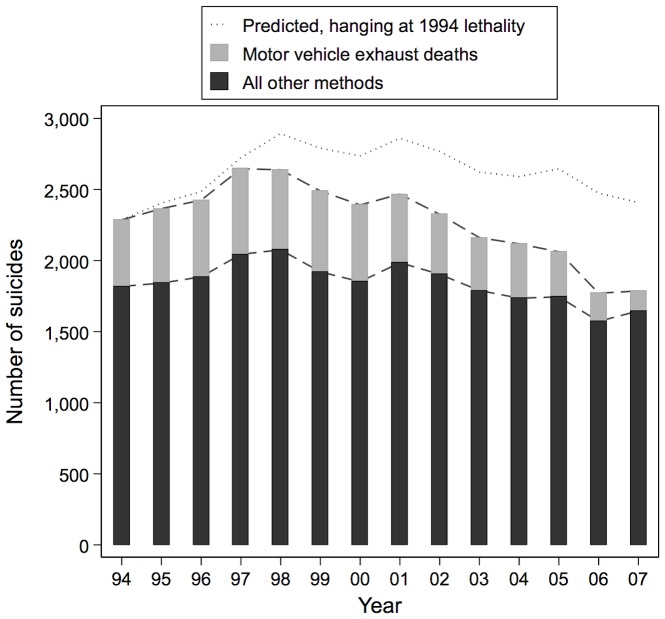
Number of suicides per year due to motor vehicle exhaust and all other methods; predicted number of suicides had lethality of hanging remained at its 1994 level.

Figure 2 showed how the steep decline in the lethality of MVE has driven substantial declines in the rate of suicide deaths by this method. **Figure 3** illustrates the influence of those MVE trends on the overall suicide death toll. The height of the bars indicate 860 fewer deaths from all methods in 2007 than in 1997; the shaded section of the bars indicate 461 fewer deaths from MVE alone over this period. Thus, the MVE decline totals 53% of the overall decline. No other single method approaches this level of impact on the national death toll.

Another way of assessing the impact of declines in the lethality of attempts on national figures is to forecast what the toll might have been had lethality not changed. This approach is particularly useful for assessing the impact of hanging, where lethality declines occurred alongside rising attempt rates. The dotted line above the bars in **Figure 3** indicates that, if hanging attempts had remained at lethal throughout as they were in 1994, the national decline in suicides would not have occurred; indeed, there would have been a small increase in the death toll.

## Discussion

This study linked the reduction in suicide deaths in Australia over the last decade to declines in the lethality of attempts by three methods: hanging, MVE and firearms. The hanging and MVE trends were particularly influential. With respect to MVE, a fairly flat incidence of attempts interacted with a 33 percentage point decrease in lethality to produce a steep decline in MVE deaths. This downward trend in MVE lethality was a major driver of the overall decline in Australia's suicide death toll. With respect to hanging, a 30-percentage point decrease in the lethality of attempts counteracted a sharp increase in the incidence of attempts. The downward trend in hanging lethality prompts consideration of what might have been: if not for the decline in the lethality of this method, the national suicide toll may have remained unchanged.

Few studies have investigated the lethality of suicide methods[8], [14], [15] and, to the best of our knowledge, only two studies have examined trends in lethality over time.[16], [17] A 2008 study[16] by Elnour and Harrison identified decreases in the lethality of three methods (hanging, motor vehicle crashes, gassing) in Australia between 1993 and 2003, but found no change in firearm lethality. This analysis provided an important clue that changes in lethality might hold a key to understanding recent suicide trends in Australia; our study was motivated in part by its findings. However, Elnour and Harrison reported limited information on time trends; the study time period ended in 2003, part way through the post-1997 decline in the national suicide rate; and, importantly, their analysis did not seek to relate changes in the lethality of particular methods to either changes in attempts or the overall suicide rate. Our study deepens understanding of changes in lethality by addressing these issues.

Why has lethality decreased for nearly all major methods of suicide? In theory, there are three possible explanations. First, reduced lethality over time may be due to factors intrinsic to the method itself, such as changes in technical features of a drug or device.[18], [19] Second, the firmness of the average attempter's intentions to die may have weakened over time (i.e. a rise in self-harm without the intention to die)[20]. Third, medical treatment for injuries due to self-harm has improved.

The first of these explanations fits for the decline in the lethality of MVE. Beginning in 1986, all new petrol-powered passenger vehicles sold in Australia were required to have catalytic converters in their engines; the same design law mandated a reduction in carbon monoxide (CO) emission levels from 24.2 g/km to 9.3 g/km.[21] A subsequent design law, phased in between 1997 and 1999, further lowered the CO limit to 2.1 g/km.[22]

Several studies[23]–[25] have linked the decline in MVE suicides in Australia to the changes in CO emission standards. Our findings support that connection. The steady decline in lethality probably tracked the gradual penetration of the newer, safer vehicles into the national fleet through natural turnover. Attempt rates rose until around 2001 and then reversed direction, likely reflecting several related developments: a growing proportion of attempts would have occurred using the cleaner post-86 and post-99 vehicles; many of those attempts would not have resulted in sufficiently serious harm to put the attempters in hospital (leaving the attempt data out of our dataset, and possibly rendering our downward trend in MVE lethality a lower bound on the true extent of decline); and prospective attempters, especially repeat attempters, may have come to learn that exhaust fumes were unlikely to cause death. This phenomenon is an example of a successful (if unintended) strategy of preventing suicide by means restriction.

By contrast, alteration to intrinsic dimensions of the method itself is not a plausible explanation for the decreases observed in the lethality of hanging. The only well-documented “breakthrough” in reducing self-harm by this method is the removal of ligature points from institutional settings.[26] However, the main impact of this reform would be expected to fall on attempt rates, not lethality; moreover, suicides in institutional settings are a minority of all deaths by hanging.[26] Other possible explanations for the decline in hanging lethality are that attempters are receiving better medical care sooner; hospitals and health departments have had a rising propensity to code admissions as injury from hanging (thereby driving up the attempt rate, and concomitantly, driving down the lethality rate in our calculations); and persons who attempted hanging did so with waning levels of intent and vigor over the study period. This last explanation is particularly intriguing, and in our view, more convincing than the other candidates, but what could have prompted such behavioral change is unclear.

Recalling that what we refer to as “attempts” would have included some acts of self-harm in which the intention was not to die, the increase in hanging attempts may be due in part to a rise in the popularity of asphyxia games (e.g. “the choking game”), particularly among youths and adolescents.[27], [28] More research is needed to explain the hanging trends–both to sift the competing explanations for the huge decline in the lethality of hanging and to identify causes of the increase in suicide attempts by this method. There is no better advertisement for importance of such research than findings from our impact analyses: without the decline in hanging lethality, the Australian suicide toll's impressive decrease of 860 deaths between 1997 and 2007 would have been completely erased.

Unlike MVE and hanging, the decline in firearm deaths over the study period was due primarily to a decline in attempts; lethality remained relatively flat. Means restriction is the most plausible explanation for the downward trend in attempts. In the wake of the Port Arthur massacre in 1996, the federal government commenced a major gun “buy-back” program, purchasing and destroying hundreds of thousands of weapons.[29], [30] Two studies have suggested an association between the buy-back and the reduction in firearm suicides, although our data suggest the reduction was driven by a decline in attempts that began before the buy-back commenced.

Our study has several limitations. First, attempt data is imperfect. The problem of over-inclusiveness, given its origins in ICD coding acts of self-harm, has already been discussed. But under-inclusiveness, based on under-ascertainment of the actual number of attempts, is also an issue. The effect of this under-ascertainment on our findings would be to bias downwards lethality statistics for all methods, especially those that tend to cause less severe injury (e.g. poisoning, MVE). However, our focus on time trends probably blunts the effect of this bias: ascertainment of attempts in hospital admissions would need to be improving substantially each year to form an alternative explanation for the decreases in lethality observed.

Second, concerns about the systematic undercounting of certain causes of death, including suicide, recently prompted the ABS to undertake revisions to its coding procedures. The revisions are expected to result in upward adjustments to the suicide death counts, although revisions will not be made to counts prior to 2007. The effects of this form of under-ascertainment on our findings are mixed. It would tend bias our lethality estimates upwards (in other words, the bias works in the opposite direction to the bias arising from under-ascertainment of attempts). Once again, however, because the undercounting is likely to have affected multiple methods and study years, its effect on the trends observed is unlikely to be substantial.

Third, our method of acquiring de-identified attempt and death data separately may have resulted in some double counting. Specifically, when an attempter was admitted to hospital and died there, the episodes may have been logged as both an attempt and a death. We could not check or correct for this overlap, but its size is likely to be trivial. Individual level data from Western Australia held for another related project allowed us to estimate the size of this overlap: 0.3% of all deaths and attempts would have been double counted in that state. A US study estimated the same type of overlap and found it to less than 0.5%[14].

Finally, we were unable to distinguish between males and females because of conditions placed on the supply of data by the data custodian. Gender differences in the epidemiology of suicide are well established. For instance, male suicide rates are consistently higher, but females have higher attempt rates.[31]

In sum, this study identified changes in method-specific lethality as a critical driver of Australia's decline in suicide deaths since 1997. The reduced lethality of MVE attempts is explicable, but unraveling the reasons behind the reduced lethality of hanging demands further research. Describing such positive trends is a valuable step toward devising strategies that can help sustain and extend them.
